# The Effect of Neem Leaf Supplementation on Growth Performance, Rumen Fermentation, and Ruminal Microbial Population in Goats

**DOI:** 10.3390/ani13050890

**Published:** 2023-02-28

**Authors:** Nittaya Taethaisong, Siwaporn Paengkoum, Walailuck Kaewwongsa, Narawich Onjai-uea, Sorasak Thongpea, Pramote Paengkoum

**Affiliations:** 1 School of Animal Technology and Innovation, Institute of Agricultural Technology, Suranaree University of Technology, Nakhon Ratchasima 30000, Thailand; 2 Program in Agriculture, Faculty of Science and Technology, Nakhon Ratchasima Rajabhat University, Nakhon Ratchasima 30000, Thailand; 3 Department of Animal Science, Faculty of Technology, Udon Thani Rajabhat University, Udon Thani 41000, Thailand

**Keywords:** neem leaf, polyethylene glycol, digestibility, growth performance, rumen fermentation, ruminal microbial population

## Abstract

**Simple Summary:**

Neem leaves are a rich source of tannin, may prevent protein degradation in the rumen, and may increase growth performance because they are natural substances; thus, they are appealing as rumen modifiers. Animal nutritionists must manipulate ruminal ecology and fermentation to improve feed intake in ruminants. Therefore, the purpose of this study was to evaluate the effect of neem leaf supplementation on digestibility, growth performance, rumen fermentation, and ruminal microbial population in goats. The findings indicated that goats fed 6% neem leaf (NL) + 15% (polyethylene glycol (PEG)) in the concentrate had the highest values of feed intake, nutrient digestion, and nitrogen utilization increased growth performance; the highest values of *Butyrivibrio fibrisolvens* and *Streptococcus gallolyticus*; and reduced protozoa and methanogens. Our results suggest that neem leaf could be an interesting substitute supplement for goat feed. We believe that neem leaf has the potential to help the goat meat industry meet the demands of health-conscious consumers.

**Abstract:**

This study aims to investigate the effect of neem leaf supplementation on the feed intake, digestibility, performance, fermentation characteristics, and ruminal microbes in goats. We included 24 Anglo-Nubian Thai native male goats with a body weight of 20 ± 2.0 kg, using 2 × 2 factorial in a completely randomized design for the following four treatments: (1) control, (2) control + 15% PEG in the concentrate, (3) 6% NL in concentrate, and (4) 6% NL + 15% PEG in concentrate. The results show that supplementation with 6% NL + 15% PEG in the concentrate had a higher (*p* < 0.05) feed intake gDM/d, % BW, g/kgBW^0.75^, nutrient intake, nutrient digestion, weight change, and ADG than did the goats that were fed with 0% NL + 0% PEG, 0% NL + 15% PEG, and 6% NL + 0% PEG in concentrate, respectively. The feeding with 6% NL + 15% PEG had a higher (*p* < 0.05) level of propionic acid at 2 and 4 h post feeding compared to the other treatments. Supplementation with 6% NL + 15% PEG in the concentrate had the lowest (*p* < 0.05) methanogen, protozoa, blood urea nitrogen, ammonia nitrogen, acetic acid, and butyric acid, as well as a lower ratio of acetic acid to propionic acid at 2 and 4 h post feeding than the other treatments. However, supplementation with 6% NL + 15% PEG in concentrate had the highest values of *Butyrivibrio fibrisolvens* and *Streptococcus gallolyticus* at 2 and 4 h post feeding compared to the other treatments (*p* < 0.05). Collectively, this study indicates that neem leaf supplements can increase growth performance and propionic acid and can modulate the abundance of *Butyrivibrio fibrisolvens* and *Streptococcus gallolyticus*. Thus, neem leaf could potentially be a good supplement for goat feed.

## 1. Introduction

Animal nutritionists are interested in improving ruminant feed efficiency by modifying rumen fermentation. Feed additives that influence rumen fermentation improve feed utilization [1]. Goat production in Thailand is increasing due to the rising demand for goat products as a result of human population growth [2]. Goats are small ruminants adapted well to the environment. They are resistant to dry weather and heat.

Neem (*Azadirachta indica*) is a tropical tree that grows in Thailand and India. The leaf extract is used as an antibacterial and bacteriostatic compound. It promotes digestibility and metabolism of proteins in ruminants. Neem leaves have been found to be a rich sources of secondary plant compounds, including alkaloids, flavonoids, polyphenolic components, and condensed tannins (CT). Moreover, they might have antimicrobial properties, particularly toward protozoal and methanogen populations. Neem leaf may prevent protein breakdown in the rumen and increase amino acid absorption in the small intestine. Animal feed containing protein–tannin compounds can increase protein production by microorganisms in goats. Tannins can decrease the bacterial population in the rumen, resulting in a decrease in the protozoa population, which could be caused by a decrease in cell membrane permeability. The effect of tannin supplementation on the methanogen population to help minimize methane permeability has been researched [3]. Tannins have a major impact in that they decrease protein-producing bacteria, including *Butyrivibrio fibrisolvens*, *Ruminobacter amylophilus*, and *Streptococcus bovis* [4]. Tannins directly inhibit methanogenic activity in the rumen by absorbing the microorganisms in the cell wall, but they also contribute to the decrease in cellulolytic bacteria in complex cellulose tannins. Fibrolytic microorganisms have a surface adhesion defect that reduces the availability of hydrogen, thereby reducing methanogens [3]. Polyethylene glycol (PEG) and polyethylene supplementation glycol are polymers capable of adhering to tannins and reducing the aggregation of protein–tannin complexes. Animals can more rapidly absorb tannins and use their ability to allow the proteins to pass into the small intestine [5]. However, the tannin and CT in neem leaves have not yet been studied for use in goats. As a result, this study was performed in order to test the following hypothesis: neem leaf feed supplements can be used in goat diets as a medicinal herb feed additive with no negative effects on dry matter, nutritional component intake, or apparent digestibility. This research also examines whether combining neem leaf with polyethylene glycol can improve feed intake, digestibility, performance, fermentation characteristics, and ruminal microbes in goats.

## 2. Materials and Methods

### 2.1. Nutrition Management

All experimental procedures used in this study were approved by the Animal Ethics Committee of Suranaree University of Technology (SUT 4/2558). All the animal experiments were performed at the Suranaree University of Technology (SUT) goat and sheep research farm, Nakhon Ratchasima, Thailand. In all, 24 Anglo-Nubian male goats with a body weight (BW) of 20 ± 2.0 kg were assigned in a 2 × 2 factorial in CRD. This research had treatment 1, control; treatment 2, control + 15% PEG in the concentrate; treatment 3, 6% NL in concentrate; and treatment 4, 6%NL + 15% PEG in the concentrate. We used pangola hay for roughage. In this experiment, we investigated whether PEG can increase CP digestibility and improve palatability. Table 1 and Table 2 display the nutrient composition of neem leaf and the chemical composition of the experimental diets. During the 60-day experiment, all goats were kept in individual feeding pens. Mineral blocks and clean water were readily available to all animals. During the 60-day experiment, the goats were fed 1.5% BW DM/day of pangola (*Digitaria eriantha*) hay and 16% crude protein in a 60:40 ratio. Those goats were provided feed that was supplemented with their specific treatment. Goats were fed at approximately 8:00 and 17:00 in the morning and afternoon, and there was a 60-day growth performance measurement period and a 7-day digestibility measurement period. The diets were formulated according to [6].

### 2.2. Feed Analyses

Throughout the experimental period, the quantity of nutrition and the quantity of refused feed were recorded on a daily basis. On the final 7 days of the experiment, feeds of given and refused feeds were collected each day. The feed (500 g) was dried for 72 h in a vacuum oven at 65 °C and ground in a Wiley mill (Retsch SM 100 mill; Retsch Gmbh, Haan, Germany) with a screen 1 mm in diameter. Chemical and nutritional tests were performed on the dried samples [7]. To obtain the crude protein (CP) values, 6.25 was used as the conversion factor. Condensed tannins were determined by HPLC [8].

### 2.3. Feces Sampling and Analyses

The total collection method was used to measure and sample daily feces during the last 7 days of each period when the animals were in metabolism cages to investigate feed digestion and nitrogen metabolism. Around 5% of the total fresh weight of fecal samples was collected and divided into two portions. The first portion was used to calculate daily dry matter (DM), while the second was stored in the refrigerator and pooled for the animals at the end of each period for chemical analysis. The method described in [9,10] was used to calculate the acid detergent fiber (ADF) and the neutral detergent fiber (NDF).

### 2.4. Urine Sampling Procedures

Urine was collected in the final 7 days at the end of the period when the animals were in metabolism cages. To evaluate N use, urinary samples were obtained at roughly 100 mL of total urine volume, kept in a refrigerator, and pooled at the end of each session. Total N was determined using AOAC procedures [11].

### 2.5. Apparent Digestibility

The apparent nutritional digestibility (%) was calculated using the acid-insoluble ash (AIA) technique as follows: apparent nutritional digestibility (%) = 100 − ((100 × % AIA in diet × % AIA in fecal)/(% AIA in fecal × % AIA in diet)) [12]. All the fecal samples were oven dried at 65 °C for 72 h crushed, passed through a 1 mm filter, and stored at 4 °C until analysis.

### 2.6. Rumen Fluid Sampling

The final day of the experiment, ruminal fluid was collected using a stomach tube connected to a vacuum pump at 0, 2, and 4 h after the animals were fed. In each case, experienced collectors collected the fluid and discarded any fluid that had saliva so as to prevent interference with pH value assessment. About 30 mL of rumen fluid was obtained at the same time as the blood sampling. Four layers of cheesecloth were used to filter the rumen fluid samples. A total of 30 mL of rumen fluid was mixed with 5 mL of sulfuric acid (H_2_SO_4_).

The rumen fluid solution was centrifuged at 16,000× *g* for 15 min, and the NH_3_-N content was determined using a Kjeltech Auto 1030 analyzer (Tecator, Hoganiis, Sweden). Total volatile fatty acid (VFA), acetate, propionate, and butyrate concentrations were determined using a Thermo Fisher Scientific Inc. (Waltham, MA, USA) analyzer.

### 2.7. Blood Sampling

A blood sample (about 5 mL) was taken from the jugular vein of each goat at 0 h, 2 h, and 4 h after feeding at 8:00 am on the final feeding week. The sample was placed in a tube (without EDTA), allowed to coagulate for 20 min at room temperature, and centrifuged for 10 min at 1107× *g* to separate the serum (Table Top Centrifuge PLC-02, Enfield, CT, USA). The collected serum was kept at −20 °C until it was analyzed (within 1 day) for blood urea nitrogen (BUN).

### 2.8. Rumen Microbial Procedures

Total DNA was collected from rumen fluid using QIAmp PowerFecal DNA kit (QIAGEN GmbH, QIAGEN Strasse 1, 0724 Hilden, Germany). A gel extraction kit (QIAGEN GmbH, QIAGEN Strasse 1, 0724 Hilden, Germany; Cat. No. 28704) was used. The purity of the extracted DNA was measured using a NanoVue spectrophotometer to measure the absorbance ratio of 260/280 (GE Healthcare Bio-Sciences, Pittsburgh, PA, USA).

The samples were tested using a Roche LightCycler^®^ 480 real-time PCR method for quantitative real-time polymerase chain reaction (PCR) amplification (Roche Diagnostics GmbH, Penzberg, Germany). There were five DNA target prime sequences: total bacteria, methanogen, protozoa, *Butyrivibrio fibrisolvens*, and *Streptococcus gallolyticus*. Real-time PCR amplifications were conducted on 10 μL reaction volume of 5 μL of 2× Roche 04707516001 LightCycler^®^ 480 SYBR Green I Master (Roche Diagnostics GmbH, Mannheim, Germany), 2 μL of 10× diluted DNA, and 1 μL of forward and reverse primers. Next, the plates (LightCycler^®^ 480 Multiwell Plate 96, white; Roche Diagnostics GmbH) were centrifuged at 4 °C at 1500 rpm for 3 min (Universal 320, Hettich Zentrifugen, Tuttlingen, Germany). The cycling conditions were as follows: the sample was kept at 95 °C for 10 min for pre-incubation, and then subjected to 40 cycles in 30s at the same temperature for amplification; the temperature was increased from 55 °C to 57.5 °C and maintained for 1 min (annealing temperature optimized based on the primer; Table 3); then, the temperature was reduced to 40 °C for cooling. For each gene, amplifications were carried out in triplicate.

### 2.9. Statistical Analysis

All the equations were evaluated as a 2 × 2 factorial in a completely randomized design (CRD). The data collected from the experiment were analyzed for variance (ANOVA), and we compared the variations between the treatments using the Statistical Analysis System 9.1.3 (SAS Inst. Inc., Cary, NC, USA) method of Duncan’s New Multiple Range Test (DMRT). According to the general model, Υijk = μ + αi + βj + (αβ)ij + ε, where Yijk denotes the observation, μ represents the overall mean, αi represents the results from the tannin that consists of i levels, βj represents the results from polyethylene glycol that consists of j levels, (αβ)ij represents the result of the sum of the tannin factor at i and the polyethylene glycol factor at j, and ε is the residual error value at k of the tannin factor at i and the polyethylene glycol factor where j is normally distributed independently and has a mean of 0 and a variance of σ 2. There are random cage for goats, and a fixed influence is dietary treatment. Significant differences were considered at *p* < 0.05.

## 3. Results

### 3.1. Feed Intake

Table 4 presents the effect of neem leaf supplementation on feed intake. There were significant differences in feed intake (gDM/d, % BW, and g/kgBW^0.75^) (*p* < 0.05) for group fed 6% NL + 15% PEG, which was the highest among all groups. This group also showed higher intake for OMI, CPI, and EE (*p* < 0.05). In this experiment, we found that neem leaf modulates feed intake without any negative influence on the animal.

### 3.2. Digestibility

Table 5 displays the data on nutrient digestion. There were significant differences (*p* < 0.05). Supplementation with 6% NL + 15% PEG increased the nutrient digestion of DDM, DOM, DCP, and DEE and reduced the nutrient digestion of DNDF and DADF.

### 3.3. Nitrogen Utilization

There were significant differences (*p* < 0.05). Supplementation with 6% NL + 15% PEG led to the highest N intake, N absorption, % N absorption, N retention, and % N retention, as shown in Table 6. Additionally, supplementation with neem leaves had no effect on N in feces and N in urine (*p* > 0.05).

### 3.4. Performance

In terms of animal growth performance (Table 7), supplementation with 6% NL + 15% PEG led to the highest final weight, weight change, and average daily gain (g/d) compared with other treatments.

### 3.5. Rumen Fermentation

Table 8 shows the effect of neem leaf in blood urea nitrogen and ammonia nitrogen in the four treatments (*p* < 0.05). Supplementation with 6% NL + 15% PEG reduced blood urea nitrogen and ammonia nitrogen at 2 and 4 h. Among goats fed neem leaf, there was no effect on pH at 0 h and 2 h; however, there was an effect on pH at 4 h.

Supplementation with 6% NL + 15% PEG affected the level of propionic acid at 2 and 4 h (*p* < 0.05), as shown in Table 9. In addition, supplementation with 6% NL + 15% PEG in the concentrate did not reduce acetic acid, butyric acid, or the ratio of acetic acid to propionic acid at 2 and 4 h after the animal was fed.

### 3.6. Microbial Population in Rumen

Table 10 shows the effect of neem leaf supplementation on rumen microbial populations. Supplementation with 6% NL + 15% PEG in the concentrate increased *Butyrivibrio fibrisolvens* and *Streptococcus gallolyticus* at 2 and 4 h post feeding (*p* < 0.05). The methanogen and protozoa values were reduced at 2 and 4 h after feeding (*p* < 0.05). However, the supplementation had no effect on total bacteria at 0 and 2 h, and reduced the total bacteria at 4 h after feeding.

## 4. Discussion

### 4.1. Feed and Nutrient Intake

The tannin-rich neem leaf has been shown to enhance the flow of rumen undegraded protein, in addition to providing nutritional benefits [13,14], because the leaves of *Leucaena* spp. plants, cassava, and Siamese neem are among the local feed sources used for livestock, including ruminant development in tropical countries, due to their high nitrogen content [13,15].

In this study, growing goats fed 6% NL + 15% PEG showed increased feed and nutrient intake. In [16,17,18,19], the authors have reported that diet containing 2–4% of tannins reduces digestion in the rumen to allow microbial proteins to pass through the small intestine and increase the absorption of essential amino acids. A study on passing of feed particles [18], which also corresponds to the study in [19], reported that rumen was not affected by tannin–yucca extracts at 8 g/d. However, this should not be used at a proportion of more than 9% in the feed, as it decreases the digestion of feed and also reduces feed intake, which can lead to death [20]. In the current study, PEG in diet increased the intake of all nutrients. Dry matter, OM, CP, EE, and ADF intakes were optimized at 15% PEG supplementation. This finding is similar to [20], which found that PEG consumption enhanced tanniniferous foliage intake and that PEG in the diet degrades tannin–fiber complexes, allowing them to be digested by microbial enzymes. The intake of dry matter by ruminant animals varies according to their size, the type of feed, the level of proteins, and fiber the animal receives, as well as the type and condition of the animal’s body and the management of the feeding process.

### 4.2. Digestibility

The results of our study once again indicated that supplementation with 6% NL + 15% PEG resulted in the highest digestibility of protein, consistent with the findings of [14]. Polyethylene glycol supplementation had no negative effects on the apparent digestibility of nutrients in the present study. The effect of neem leaf on the digestion, modifying the ruminal microbes and development of tannin–protein complexes, has been well established in ruminant diets [21,22]. The addition of neem leaf in ruminant formulas for chewing was found to create a compound of tannin and digestive saliva. The use of condensed tannin from *Lotus corniculatus* in the late stage of milking feeding was found to increase milk production and milk proteins [23], increase the microbial proteins released from the rumen [24,25,26,27], and increase wool production in sheep [26]. Ref. [28] reported that PEG 4000 supplementation did not affect NDF and ADF digestibility in Pedi goats fed *Acacia nilotica* leaf meal. According to [28], PEG supplementation significantly improved the apparent digestibility of nutrients. It can be used to feed animals, and tannins can prevent digestion in the rumen and increase the protein microbes passing through the intestines. This in turn can increase the absorption of essential amino acids because condensed tannins in forage protein can replace proteins that are not digested in the rumen from other sources. However, they should not be added at a proportion of more than 9% in dry-matter feed, as this will reduce the feed intake and may lead to the animal’s death [20].

### 4.3. Performance

Studies have shown that supplementation with 6% NL + 15% PEG resulted in the highest weight change and ADG. The optimal amount of concentrated tannins for animal feed use is 2–4% in the concentrate, which can prevent digestion in the rumen, increase the amount of microorganisms and proteins that pass through the small intestine, and also increase the absorption of essential amino acids. They can also decrease the incidence of both [27,29]. However, large doses may harm animals, in particular their reproductive and physiological systems. For example, changes in the dietary rates result in changes in the rate of feeding, digestion, growth rate, and movement of the rumen, and these can affect microbe function. Some studies have indicated that tannins exceeding 9% should not be added to the feed, as this can contribute to the death of the animal due to reduced assimilation via rumen digestion and decreased levels of nitrogen in the body [20,30].

In this study, supplementation with 6% NL + 15% PEG in the concentrate led to the highest performance in goats, even at high condensed tannin concentrations. In addition, the recorded amounts had no adverse effects on the animals and were able to increase the efficiency of the goats because the polyethylene glycol in the concentrate combined with the tannins and decreased toxic tannins. Polyethylene glycol reduces the effectiveness of tannins. The addition of polyethylene glycol (PEG) to tannin-rich diets is another attractive alternative for enhancing the feeding value of such diets. Because PEG has a stronger affinity for tannins than proteins, it is thought to break tannin–protein structures. This product has been used to counteract the effects of tannins [31].

### 4.4. Rumen Fermentation Parameters

Supplementation with 6% NL + 15% PEG in the concentrate had no effect on nutrient digestion or nitrogen excretion. Neem leaf has been shown to prevent digestion in the rumen and increase the amounts of nitrogen that accumulate and circulate in the body. Although condensed tannins can decrease the feed intake of animals, the optimal amount does not affect feed intake. The levels of condensate in the tannins influence the nitrogen levels in the bodies of dairy cows [31,32]. The amount in the form of dry matter should not be more than 5% of the feed, which will provide benefits because of reduced digestion in the rumen. However, if more than 5% is used in the feed, the digestion in the rumen will increase, and the levels of nitrogen in the body will decrease.

Supplementation with 6% NL + 15% PEG in the concentrate reduced the blood urea concentration. The blood urea nitrogen level indicates the mechanism of protein change in ruminant animals, related to the ammonia nitrogen in the rumen liquid. However, the values of blood urea nitrogen differ depending on, for example, the amount of protein received by the animal for digestion, the level of energy, and the oxidation of proteins in the body to produce energy during fasting, including amino acids that are not used in protein synthesis, which are converted into blood urea nitrogen. The amount of protein that animals obtain from feed may be influenced by high blood urea nitrogen levels [3]. Supplementation with 6% NL + 15% PEG resulting in the highest pH value (6.52) revealed that there was no impact due to the use of neem as a source of polyethylene glycol condensate. The optimal pH value in the rumen is between 6.5 and 7.0, which is ideal for the growth of microorganisms in the rumen [3]. The pH value of the rumen was within the appropriate range and suitable for microbial growth in the rumen. Moreover, tannins have an acidic influence, but the protein–tannin compounds were found to reduce rumen pH. In animal feed containing polyethylene glycol tannins, polyethylene glycol tends to decrease the potency of tannins, which means that this experiment used condensed tannins at amounts higher than the acceptable standard. In this research, the neem leaf did not harm the animals because we used polyethylene glycol with condensed neem tannin, a polyethylene combination that helps minimize the toxicity of condensed tannins. PEG improves the consumption of tannin-containing feeds by acting as a tannin binding agent without changing the genetic pool of tannin-containing plants.

Supplementation with 6% NL + 15% PEG led to the lowest NH_3_-N levels at 2 h (11.33 mg/dL) in the rumen, and it is a nitrogen source essential for growth in the rumen in this experiment. This is because the effect of CTs on digestion by modifying the population of ruminal microbes and tannin–protein complexes has been well established in terms of reducing ruminal crude protein degradation in ruminants, leading to reduced ruminal NH_3_-N concentration [14]. In [33], the optimum NH_3_-N levels were found to be in the range of 9.7–21.4 mg/dL. The study in [34] stated that 17.6 mg/dL was the acceptable amount. Increased digestibility of dry matter, protein, and bacterial communities in the rumen results from this stage. The predetermined range of PEG with tannin over protein saves the protein for rumen fermentation, resulting in higher NH_3_-N levels in rumen fluid. The amount of ammonia nitrogen, which is related to the level of nitrogen in the bloodstream, also increases in the rumen, leading to increased blood urea nitrogen.

Supplementation with 6% NL + 15% PEG resulted in high values of propionic acid at 2 and 4 h and a decrease in total VFA, but with values within the normal range for goats. Because of its high molecular weight, neem leaf has a strong effect on total VFAs and acetic acid production, unlike low molecular weight substances [13]. Since tannins can bind to enzymes, especially in cellulose, the decrease in total VFAs caused by condensed tannin supplementation may be due to a reduction in microbial activity [13]. The ratio of acetic acid to propionic acid, both before and after the use of neem leaf, was found to have no adverse effect on goat disease in this experiment. Furthermore, the condensed tannin-affected decrease in acetic acid and the increase in propionic acid proportions indicate that nutrients were partitioned more into microbial protein synthesis [13]. Higher VFA production in 15% PEG is caused by particular tannin binding with PEG, which leads to enhanced fermentation.

### 4.5. Microbial Population in Rumen

Supplementation with 6% NL + 15% PEG is reported to have antibacterial properties, helping to reduce gastrointestinal tract fermentation. In this study, the use of neem leaf in the concentrate led to the highest amount of tannins, which were found to increase *Butyrivibrio fibrisolvens* and *Streptococcus gallolyticus* at 2 and 4 h. It was also discovered that *Streptococcus gallolyticus* can assist animal health by helping improve goat growth and reducing the occurrence of mastitis. Neem leaf supplementation can reduce the quantity of *Streptococcus gallolyticus*, [35], increase the use of nutrients, and prevent mastitis. The study in [36] found that *Streptococcus gallolyticus*, which improves the digestibility of nitrogen in sheep, may be reduced due to supplementation with tannins. The rumen bacterium *Butyrivibrio fibrisolvens* has been identified as undertaking biohydrogenation of fatty acids and forming conjugated linoleic acid (CLA), which is an intermediate isomer of C18:2, in the process.

In this study, supplementation with 6% NL + 15% PEG reduced methane production because neem leaf contains tannins, compounds with good anti-protozoa properties. Adding condensed tannins with a higher molecular weight has been found to reduce acetic acid formation and CH_4_ production [36,37]. Tannins enter through the cell membrane and attack the structure of the protozoa cell membrane because the cell membrane covers the entire inner portion consisting of fat and protein layers [38], which improves rumen fermentation and may increase high-protein microbes.

In this experiment, supplementation with 6% NL + 15% PEG increased *Butyrivibrio fibrisolvens* at 2 and 4 h, which increased the amount of protein-producing microorganisms. Nevertheless, depending on the tannin and microbial levels in the rumen, roughage is one of the factors that affects the rumen microbe population. In [39], it was reported that feed concentrate with a high tannin content and tannins that bind to proteins resulted in complex structures that may affect the microbes that digest fiber and reduce the digestibility of proteins in the rumen.

## 5. Conclusions

In our study, supplementation with 6% NL + 15% PEG in the concentrate increased the feed intake, nutrient intake, nutrient digestion, nitrogen utilization, and growth performance of goats. Moreover, supplementation with 6% NL + 15% PEG in the concentrate had the highest propionic acid, *Butyrivibrio fibrisolvens*, and *Streptococcus gallolyticus*, as well as decreased protozoa and methanogens. Further studies are warranted to investigate the effect of neem leaf on meat products and antioxidant activity in meat.

## Figures and Tables

**Table 1:** Chemical composition of neem leaf.

Ingredient	Neem Leaf
Chemical Composition (% DM)	
Dry matter	36.00
Crude protein	18.83
Ash	7.78
Ether extract	1.52
Non-fibrous carbohydrate	30.15
Neutral detergent fiber	41.72
Acid detergent fiber	31.52
% Condensed tannin	10.66

**Table 2:** Diets’ nutrition and composition.

Diet						
Items	0% NL + 0% PEG	0% NL + 15% PEG	6% NL + 0% PEG	6% NL + 15% PEG	SEM	*p*-Value
Soybean meal	17.10	15.57	15.00	12.20		
Rice bran	30.00	24.33	25.00	22.01		
Cassava chip	22.00	25.00	25.40	22.61		
Corn	29.80	19.00	27.50	21.08		
Sodium chloride	0.40	0.40	0.40	0.40		
Pure sulfur	0.20	0.20	0.20	0.20		
Minerals and vitamins	0.50	0.50	0.50	0.50		
Condensed tannin	0.00	0.00	6.00	6.00		
Polyethylene glycol	0.00	15.00	0.00	15.00		
Chemical composition (% DM)						
Dry matter	74.05	74.43	74.07	74.05	0.05	0.01
Ash	6.16	5.99	6.77	6.43	0.09	0.01
Crude protein	16.30	16.45	16.55	16.65	0.04	0.01
Ether extract	1.34	2.01	2.13	2.25	0.11	0.01
Non-fibrous carbohydrate	23.73	24.7	16.56	19.51	0.99	0.01
Neutral detergent fiber	52.47	50.85	57.99	55.16	0.82	0.01
Acid detergent fiber	24.23	23.65	32.21	31.40	1.19	0.01
TDN, %	88.70	88.64	87.20	87.15	0.23	0.01
Metabolizable energy, Mcal/kg DM	3.21	3.20	3.15	3.15	0.01	<0.01

NL = neem leaf, PEG = polyethylene glycol.

**Table 3:** Primer sequences used in this analysis for real-time PCR amplification.

Items	Forward/ Reverse	Temperature (°C)	Product Size (bp)	Primer Sequence (5′-3′)
Total bacteria	F	55	130	CGGCAACGAGCGCAACCC
	R			CCATTGTAGCACGTGTGTAGCC
Methanogen	F	58	140	TTCGGTGGATCDCARAGRGC
	R			GBARGTCGWAWCCGTAGAATC
Protozoa	F	55	223	CTTGCCCCTCYAATCGTWCT
	R			GCTTTCGWTGGTAGTGTATT
*Butyrivibrio fibrisolvens*	F	58	64	ACACACCGCCCGTCACA
	R			TCCTTACGGTTGGGTCACAGA
*Streptococcus gallolyticus*	F	58	419	GAAAAGTACTCAACCAAATA
	R			AGTAACGGTACTTAAATTGTTTA

**Table 4:** Effect of neem leaf supplementation on feed intake in goats.

	0% NL		6% NL				*p*-Value	
TRT	0% PEG	15% PEG	0% PEG	15% PEG	SEM	NL	PEG	NL × PEG
Feed intake								
gDM/d	456.84 ^d^	469.91 ^c^	476.96 ^b^	495.87 ^a^	0.57	0.44	0.44	<0.01
% BW	2.11 ^c^	2.11 ^c^	3.95 ^b^	4.07 ^a^	0.20	0.44	0.46	0.01
g/kgBW^0.75^	41.94 ^d^	44.46 ^c^	66.53 ^b^	75.41 ^a^	2.95	0.44	0.45	0.02
Nutrient intake g DM/d								
OMI	429.43 ^d^	441.72 ^c^	443.57 ^b^	466.12 ^a^	0.39	0.0015	0.02	<0.01
CPI	73.09 ^d^	75.19 ^c^	81.08 ^b^	84.30 ^a^	0.25	0.01	0.01	0.04
EEI	4.56 ^d^	9.40 ^c^	9.54 ^b^	9.92 ^a^	0.16	0.01	0.01	0.01
NDFI	237.56 ^d^	239.65 ^c^	276.64 ^a^	272.23 ^b^	0.93	0.01	0.01	0.01
ADFI	109.64 ^d^	112.78 ^c^	152.63 ^b^	153.72 ^a^	0.58	0.01	0.01	0.01

NL, neem leaf; PEG, propylene ethylene glycol; SEM, standard error of the mean; g DM/d, daily intake of dry matter; a, b, c, and d, in the same row, indicate statistically significant difference *p* < 0.05. %BW, ((g DM intake × 100)/ (body weight (kg) × 1000)); g/kgBW^0.75^, g DM intake/ (kg body weight)^0.75^.

**Table 5:** Effect of neem leaf supplementation on nutrient digestion in goats.

	0% NL		6% NL				*p*-Value	
TRT	0% PEG	15% PEG	0% PEG	15% PEG	SEM	NL	PEG	NL × PEG
Apparent Digestibility, % of intake								
DDM	72.80 ^d^	76.83 ^c^	79.85 ^b^	82.03 ^a^	0.76	0.44	0.46	0.08
DOM	75.97 ^b^	76.68 ^b^	81.45 ^a^	82.39 ^a^	0.63	0.44	0.09	0.81
DCP	40.81	33.03	45.49	60.70	0.32	0.47	0.66	0.42
DEE	79.99 ^c^	82.18 ^b^	85.95 ^a^	85.55 ^a^	0.58	0.45	0.12	0.03
DNDF	31.89 ^a^	33.32 ^c^	35.04 ^b^	32.58 ^d^	0.51	0.0076	0.46	0.08
DADF	21.78 ^c^	23.16 ^b^	26.34 ^a^	21.04 ^c^	0.47	0.0224	0.0006	0.01

NL, neem leaf; PEG, propylene ethylene glycol; SEM, standard error of the mean; DDM, digestibility of dry matter; DOM, digestibility of organic matter; DCP, protein digestibility; DEE, fat digestibility; DEE, digestibility fatty digestibility; DNDF, the digestibility of the crude fiber that cannot be dissolved in neutral solution; DADF, the digestibility of the fiber that cannot be digested in an acidic solution; a, b, c, and d, in the same row, indicate statistically significant difference *p* < 0.05.

**Table 6:** Effect of neem leaf supplementation on nitrogen utilization in goats.

	0% NL		6% NL				*p*-Value	
TRT	0% PEG	15% PEG	0% PEG	15% PEG	SEM	NL	PEG	NL × PEG
N intake (g/d)	36.39 ^c^	26.64 ^d^	37.59 ^b^	45.72 ^a^	1.43	0.44	0.23	<0.01
N feces (g/d)	21.54	17.84	20.49	17.97	0.37	0.30	0.45	0.17
N urine (g/d)	5.47	5.38	5.78	5.38	0.06	0.20	0.04	0.17
N absorption (g/d)	14.85 ^c^	8.80 ^d^	17.1 ^b^	27.75 ^a^	0.85	0.66	0.0014	0.28
N absorption (%)	40.81 ^c^	33.03 ^d^	45.49 ^b^	60.70 ^a^	1.02	0.66	0.001	0.28
N retention (g/d)	9.38 ^c^	3.42 ^d^	11.32 ^b^	22.37 ^a^	0.63	0.44	0.04	0.02
N retention (%)	25.78^c^	12.84 ^d^	30.11 ^b^	48.93 ^a^	0.48	0.0004	0.02	0.07

NL, neem leaf; PEG, polyethylene glycol; SEM, standard error of the mean; a b c, d, in the same row there are significant differences in Statistics (*p* < 0.05).

**Table 7:** Effect of neem leaf supplementation on performance.

	0% NL		6% NL				*p*-Value	
TRT	0% PEG	15% PEG	0% PEG	15% PEG	SEM	NL	PEG	NL × PEG
Initial weight, kg	20.33	20.67	20.83	21.00	0.15	0.19	0.43	0.79
Final weight, kg	24.00	24.18	26.00	26.54	0.27	0.03	0.68	0.20
Weigh change, kg	3.67 ^c^	3.51 ^d^	5.17 ^b^	5.54 ^a^	0.19	0.06	0.44	0.31
ADG, g/d	40.78 ^c^	39.00 ^d^	57.44 ^b^	61.56 ^a^	3.42	0.44	0.12	0.46

NL, neem leaf; PEG = level of polyethylene glycol; SEM, standard error of the mean; a, b, c, and d, in the same row there are significant differences in Statistics (*p* < 0.05).

**Table 8:** Effect of neem leaf supplementation on pH, ammonia nitrogen, and blood urea nitrogen.

	0% NL		6% NL				*p*-Value	
TRT	0% PEG	15% PEG	0% PEG	15% PEG	SEM	NL	PEG	NL × PEG
BUN Mg%								
0 h	23.03	21.23	17.66	20.09	0.56	0.0022	0.73	0.03
2 h	17.82 ^a^	14.81 ^b^	14.89 ^b^	12.97 ^c^	0.45	0.0002	0.47	0.29
4 h	18.20 ^a^	17.82 ^b^	15.88 ^c^	14.44 ^d^	0.40	0.46	0.06	0.25
Mean	19.79 ^a^	17.90 ^b^	16.11 ^c^	15.82 ^c^	0.26	0.01	0.007	0.04
Ruminal pH								
0 h	6.76	6.67	6.74	6.90	0.04	0.27	0.66	0.17
2 h	6.58	6.53	6.69	6.68	0.03	0.06	0.59	0.71
4 h	6.43 ^b^	6.24 ^d^	6.32 ^c^	6.52 ^a^	0.02	0.44	0.58	<0.01
Mean	6.59 ^ab^	6.48 ^c^	6.58 ^cb^	6.70 ^a^	0.03	0.01	0.94	<0.01
Ruminal NH_3_-N mg/dL								
0 h	16.92	14.24	14.79	14.6	0.43	0.23	0.09	0.13
2 h	15.08 ^a^	13.49 ^c^	14.23 ^b^	11.33 ^d^	0.31	0.0003	0.45	0.06
4 h	12.10	13.91	12.29	10.09	0.41	0.01	0.77	<0.01
Mean	14.59 ^a^	13.80 ^a^	13.77 ^a^	12.00 ^b^	0.33	0.01	0.01	0.30

NL, neem leaf; PEG, polyethylene glycol; BUN, concentration of urea in the blood; NH_3_-N, ammonia nitrogen; SEM, standard error of the mean; a, b, c, d, in the same row there are significant differences in Statistics (*p* < 0.05).

**Table 9:** Effect of neem leaf supplementation on volatile fatty acids in goats.

	0% NL		6% NL				*p*-Value	
TRT	0% PEG	15% PEG	0% PEG	15% PEG	SEM	NL	PEG	NL × PEG
Acetic acid (% Molar)								
0 h	62.22	61.39	60.49	60.80	5.40	0.44	0.65	0.67
2 h	53.14 ^c^	56.66 ^a^	54.06 ^b^	53.48 ^c^	0.65	0.45	0.45	<0.01
4 h	53.69	50.56	52.51	51.14	0.32	0.04	0.47	0.71
Mean	50.20	45.85	46.19	45.12	1.30	0.22	0.16	0.37
Propionic acid (% Molar)								
0 h	22.21	26.41	26.19	28.07	1.48	0.59	0.31	0.85
2 h	25.08 ^d^	26.06 ^c^	29.99 ^b^	32.13 ^a^	0.47	0.45	0.06	<0.01
4 h	22.96 ^d^	27.51 ^b^	26.51 ^c^	28.51 ^a^	0.48	0.46	0.46	<0.01
Mean	21.45 ^c^	22.38 ^bc^	23.56 ^ab^	24.70 ^a^	0.34	0.0003	0.05	0.82
Butyric acid (% Molar)								
0 h	15.57	12.20	13.33	11.13	3.41	0.56	0.43	0.78
2 h	21.78 ^a^	12.28 ^d^	15.96 ^b^	14.39 ^c^	0.80	0.45	0.45	<0.01
4 h	23.36 ^a^	21.92 ^b^	20.92 ^c^	20.35 ^c^	0.25	0.01	0.06	0.95
Mean	18.25 ^a^	14.22 ^b^	14.03 ^b^	12.82 ^b^	0.80	0.03	0.03	0.22
Ratio of acetic acid to propionic acid								
0 h	2.80	2.32	2.31	2.17	0.23	0.40	0.84	0.99
2 h	2.12 ^a^	2.17 ^b^	1.80 ^a^	1.66 ^b^	0.01	0.62	0.44	0.12
4 h	2.34 ^a^	2.84 ^b^	1.98 ^c^	1.79 ^d^	0.03	0.44	0.44	0.63
Mean	2.27 ^a^	2.18 ^ab^	2.10 ^ab^	2.01 ^b^	0.05	0.05	0.25	0.99
Total VFA (mmol/L)								
0 h	83.57	76.98	75.04	74.32	3.96	0.94	0.22	0.66
2 h	96.64 ^a^	79.98 ^c^	82.73 ^b^	82.82 ^b^	1.45	0.44	0.45	<0.01
4 h	87.56 ^c^	89.01 ^b^	91.71 ^a^	89.62 ^b^	0.45	0.01	0.04	0.72
Mean	82.02 ^b^	80.62 ^b^	83.49 ^ab^	86.82 ^a^	1.01	0.02	0.51	0.11

NL, neem leaf; PEG, polyethylene glycol; VFA, volatile fatty acids; SEM, standard error of the mean; a, b, c, and d, in the same row there are significant differences in Statistics (*p* < 0.05).

**Table 10:** Effect of neem leaf supplementation on rumen microbial populations in goats.

		0% NL		6% NL				*p*-Value	
TRT		0% PEG	15% PEG	0% PEG	15% PEG	SEM	NL	PEG	NL × PEG
Total bacteria (lg10 copies/mL)	0 h	8.66	7.09	8.87	8.97	0.90	0.61	0.71	0.67
	2 h	10.57	10.64	10.53	10.70	0.03	0.83	0.03	0.34
	4 h	10.35 ^d^	10.74 ^a^	10.53 ^c^	10.63 ^b^	0.04	0.67	0.01	0.04
	Mean	9.86	9.49	9.98	10.10	0.46	0.59	0.85	0.70
Methanogen (lg10 copies/mL)	0 h	7.71	7.45	7.64	7.36	0.06	0.51	0.03	0.90
	2 h	7.40 ^b^	7.50 ^a^	7.58 ^a^	7.34 ^c^	0.02	0.81	0.02	<0.01
	4 h	7.42 ^a^	7.30 ^b^	7.20 ^c^	7.16 ^d^	0.02	0.44	0.47	0.03
	Mean	7.51 ^a^	7.42 ^a^	7.47 ^a^	7.29 ^b^	0.03	0.05	0.003	0.23
Protozoa (lg10 copies/mL)	0 h	4.85 ^c^	6.30 ^a^	5.47 ^b^	3.11 ^d^	1.76	0.19	0.63	0.05
	2 h	6.58 ^a^	5.43 ^b^	6.65 ^a^	4.66 ^c^	0.17	0.46	0.44	<0.01
	4 h	7.35 ^a^	5.35 ^b^	4.39 ^c^	2.34 ^d^	4.90	0.44	0.44	0.80
	Mean	9.32 ^a^	5.72 ^b^	5.40 ^b^	3.59 ^b^	0.84	0.02	0.03	0.45
*Butyrivibrio fibrisolvens* (lg10 copies/mL)	0 h	8.17	8.45	8.37	8.74	0.26	0.37	0.21	0.87
	2 h	7.95 ^c^	8.13 ^b^	8.25 ^b^	9.27 ^a^	0.03	0.44	0.44	<0.01
	4 h	7.99 ^c^	8.07 ^b^	8.23 ^b^	9.25 ^a^	0.02	0.44	0.44	<0.01
	Mean	8.04 ^b^	8.22 ^ab^	8.30 ^ab^	8.41 ^a^	0.06	0.02	0.10	0.67
*Streptococcus gallolyticus* (lg10 copies/mL)	0 h	8.66	7.09	8.87	8.97	0.90	0.61	0.71	0.67
	2 h	10.56 ^b^	10.56 ^b^	10.58 ^b^	11.64 ^a^	0.01	0.44	0.44	<0.01
	4 h	10.55 ^b^	10.36 ^b^	10.53 ^b^	11.61 ^a^	0.01	0.44	0.44	<0.01
	Mean	9.86	9.45	10.00	10.02	0.47	0.60	0.77	0.74

NL, neem leaf; PEG, propylene glycol; SEM, standard error of the mean; a, b, c, and d, in the same row there are significant differences Statistics (*p* < 0.05).

## Data Availability

Not applicable.
